# Neurotoxic kynurenine metabolism is increased in the dorsal hippocampus and drives distinct depressive behaviors during inflammation

**DOI:** 10.1038/tp.2016.200

**Published:** 2016-10-18

**Authors:** J M Parrott, L Redus, D Santana-Coelho, J Morales, X Gao, J C O'Connor

**Affiliations:** 1 Department of Pharmacology, School of Medicine, University of Texas Health Science Center at San Antonio, San Antonio, TX, USA; 2 Center for Biomedical Neuroscience, University of Texas Health Science Center at San Antonio, San Antonio, TX, USA; 3 Department of Biochemistry, School of Medicine, University of Texas Health Science Center at San Antonio, San Antonio, TX, USA; 4 Audie L. Murphy Memorial VA Hospital, South Texas Veterans Health System, San Antonio, TX, USA

## Abstract

The kynurenine pathway of tryptophan metabolism has an important role in mediating the behavioral effects of inflammation, which has implications in understanding neuropsychiatric comorbidity and for the development of novel therapies. Inhibition of the rate-limiting enzyme, indoleamine 2,3-dioxygenase (IDO), prevents the development of many of these inflammation-induced preclinical behaviors. However, dysregulation in the balance of downstream metabolism, where neuroactive kynurenines are generated, is hypothesized to be a functionally important pathogenic feature of inflammation-induced depression. Here we utilized two novel transgenic mouse strains to directly test the hypothesis that neurotoxic kynurenine metabolism causes depressive-like behavior following peripheral immune activation. Wild-type (WT) or kynurenine 3-monooxygenase (KMO)-deficient (KMO^−/−^) mice were administered either lipopolysaccharide (LPS, 0.5 mg kg^−1^) or saline intraperitoneally. Depressive-like behavior was measured across multiple domains 24 h after immune challenge. LPS precipitated a robust depressive-like phenotype, but KMO^−/−^ mice were specifically protected from LPS-induced immobility in the tail suspension test (TST) and reduced spontaneous alternations in the Y-maze. Direct administration of 3-hydroxykynurenine, the metabolic product of KMO, caused a dose-dependent increase in depressive-like behaviors. Mice with targeted deletion of 3-hydroxyanthranilic acid dioxygenase (HAAO), the enzyme that generates quinolinic acid, were similarly challenged with LPS. Similar to KMO^−/−^ mice, LPS failed to increase immobility during the TST. Whereas kynurenine metabolism was generally increased in behaviorally salient brain regions, a distinct shift toward KMO-dependent kynurenine metabolism occurred in the dorsal hippocampus in response to LPS. Together, these results demonstrate that KMO is a pivotal mediator of hippocampal-dependent depressive-like behaviors induced by peripheral LPS challenge.

## Introduction

Although it remains a major health burden worldwide, the etiology of depression remains unclear. Recent studies indicate that pro-inflammatory cytokines may contribute to the development of depression,^1^ particularly as a comorbidity. Patients undergoing cytokine immunotherapy^2^ or healthy volunteers who receive immune activating agents, such as endotoxin^3, 4^ or vaccines^5, 6^ in an experimental setting, report developing depression symptoms spanning multiple dimensions (for example, mood, anhedonia, cognition, neurovegetative and anxiety). The time course and severity of these symptoms are positively correlated with pro-inflammatory cytokine levels.^4, 5^ Consistent with these clinical observations, peripheral immune activation in rodent models precipitates pro-inflammatory cytokine-dependent^7, 8, 9^ depressive- and anxiety-like behaviors.

**Figure 1:**
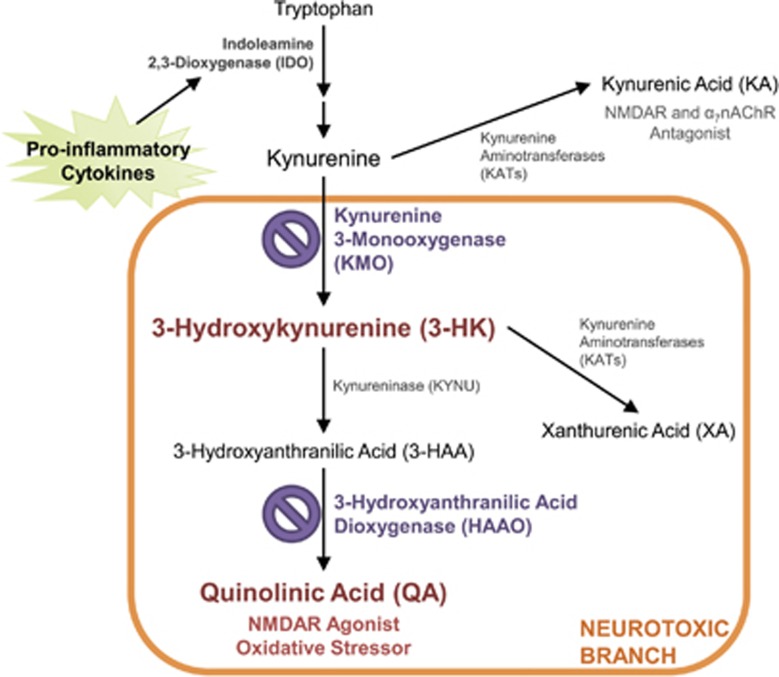
The kynurenine pathway of tryptophan metabolism. In the kynurenine pathway, tryptophan is metabolized to kynurenine by indoleamine 2,3-dioxygenase (IDO), an enzyme that is upregulated by pro-inflammatory cytokines. Kynurenine is then either metabolized to kynurenic acid (KA) by kynurenine aminotransferases (KATs), or to 3-hydroxykynurenine (3-HK, red) by kynurenine-3-monooxygenase (KMO, purple). Under basal conditions, most of kynurenine in the brain is metabolized to KA, a *N*-methyl-D-aspartate (NMDA) and α_7_-nicotinic acetylcholine (α_7_nACh) receptor antagonist. However, neuroinflammation and pro-inflammatory cytokines will shift kynurenine metabolism through KMO to 3-HK. Then, further metabolism occurs to 3-hydroxyanthranilic acid (3-HAA) by kynureninase (KYNU), and then 3-HAA is metabolized to quinolinic acid (QA, red) by 3-hydroxyanthranilic acid dioxygenase (HAAO, purple). During neuroinflammation, QA is the major end product of the kynurenine pathway, a metabolite that is a NMDA receptor agonist and an oxidative stressor. 3-HK can also be metabolized to xanthurenic acid (XA) by KATs when substrate levels are high enough. This branch of metabolites (3-HK, 3-HAA, QA, XA) is considered to be neurotoxic (orange box) as they can contribute to oxidative stress and glutamate excitotoxicity. In the studies described, two genetic mouse models were used (KMO and HAAO knockouts) to target neurotoxic kynurenine metabolism (indicated by purple ‘no' symbol).

Peripheral pro-inflammatory cytokines signal to the brain via multiple routes^10^ and activate the resident immune cells of the brain, microglia. Activated microglia, in turn, secrete cytokines and chemokines, generate oxidative stress molecules and alter local metabolic processes.^11^ Disruption in the balance of tryptophan metabolism along the kynurenine pathway is a putative mechanism linking inflammation, microglia and depression. The rate-limiting enzyme that metabolizes tryptophan to kynurenine, indoleamine 2,3-dioxygenase (IDO, Figure 1), is potently upregulated by pro-inflammatory cytokines.^12^ Inflammation-associated depression scores in human patients are associated with an elevated kynurenine/tryptophan ratio, an indicator of IDO activity,^13^ and numerous preclinical mouse models have established IDO-dependent kynurenine metabolism as an important mediator of inflammation-induced depressive-like behaviors.^14, 15, 16, 17, 18^ Interestingly, microglia are the predominant cells expressing the enzyme (kynurenine 3-monooxygenase, KMO, Figure 1) for the generation of neurotoxic kynurenine metabolites, and reports in both human^19^ and in mice^20^ have implicated microglial-derived^21, 22^ downstream neurotoxic kynurenine metabolites in the pathogenesis of inflammation-associated depression; however, mechanistic studies are yet to be performed.

Kynurenine metabolism in the brain results in accumulation of two major neuroactive end products, kynurenic acid (KA) and quinolinic acid (QA; Figure 1). Under basal conditions, most kynurenine is metabolized by astrocytes to KA, an *N*-methyl-D-aspartate (NMDA) and α_7_-nicotinic acetylcholine receptor antagonist.^23, 24^ However, inflammation and subsequent microglial activation is reported to shift kynurenine metabolism toward KMO-dependent production of QA.^20, 25^ QA, an NMDAR agonist, can be particularly disruptive to the neuronal environment at higher concentrations, not only by elevating the potential for glutamate excitotoxicty but also by precipitating oxidative damage^26^ and potentiating pro-inflammation. A neurotoxic shift in kynurenine metabolism favoring the production of KMO-dependent metabolites is hypothesized to mediate inflammation-associated behavior changes.^27^ Increased levels of cerebrospinal fluid (CSF) QA in patients undergoing interferon-α immunotherapy were positively correlated to more severe depression scores.^19^ A similar elevation in central QA and its neurotoxic precursor, 3-hydroxykynurenine (3-HK), was measured in mice injected peripherally with lipopolysaccharide (LPS). Importantly, LPS-challenged mice also exhibit depressive- and anxiety-like behaviors concomitant with the elevations in neurotoxic metabolites.^20^ Whereas evidence implicates KMO-dependent kynurenine metabolism as a likely pathogenic mechanism underlying inflammation-induced depression,^28^ until recently, no preclinical genetic models were available to directly test this hypothesis and brain-penetrant KMO inhibitors are not commercially available.

In order to determine whether KMO-dependent neurotoxic kynurenine metabolism mediates the depressive-like behavioral changes following peripheral immune challenge, two novel transgenic mouse strains were generated with targeted deletion of either the KMO or 3-hydroxyanthranilic acid dioxygenase (HAAO) gene. Inflammation-induced depressive behaviors were precipitated in control or transgenic mice using the well-established peripheral LPS challenge model.^29, 30^ 3-HK, the initial substrate for neurotoxic kynurenine pathway metabolism, was directly administered to naive mice to determine the behavioral consequences of increased KMO-dependent kynurenine metabolites. Because it had previously only been investigated at the whole-brain level, changes in downstream neuroactive kynurenine metabolism in behaviorally salient discrete brain regions were characterized in wild-type (WT) mice following LPS challenge. These novel data have important ramifications for not only our understanding of neuropsychiatric pathology during inflammation, but also for identification of novel therapeutic targets.

## Materials and methods

### Animals

All animal care and use was carried out in accord with the Guide for the Care and Use of Laboratory Animals, 8th edition (NRC) and approved by the Institutional Animal Care and Use Committee at The University of Texas Health Science Center at San Antonio. General health of the mice was monitored daily. Knockout first, conditional ready KMO and HAAO transgenic mice (Kmo^tm1a(KOMP)Wtsi^ and Haao^tm1a(KOMP)Wtsi^) on a C57BL/6N background were designed and generated by the Mouse Biology Program (MBP, www.mousebiology.org) at the University of California Davis (UC Davis). The MBP was supported by the Knock-Out Mouse Project (KOMP), a trans-NIH initiative, and the CSD Consortium, composed of the Children's Hospital Oakland Research Institute (CHORI), the Wellcome Trust Sanger Institute and UC Davis. Vectors, embryonic stem cells and gene information related to these mouse strains are available at the KOMP Repository (www.komp.org) maintained by UC Davis and CHORI.

**Figure 2:**
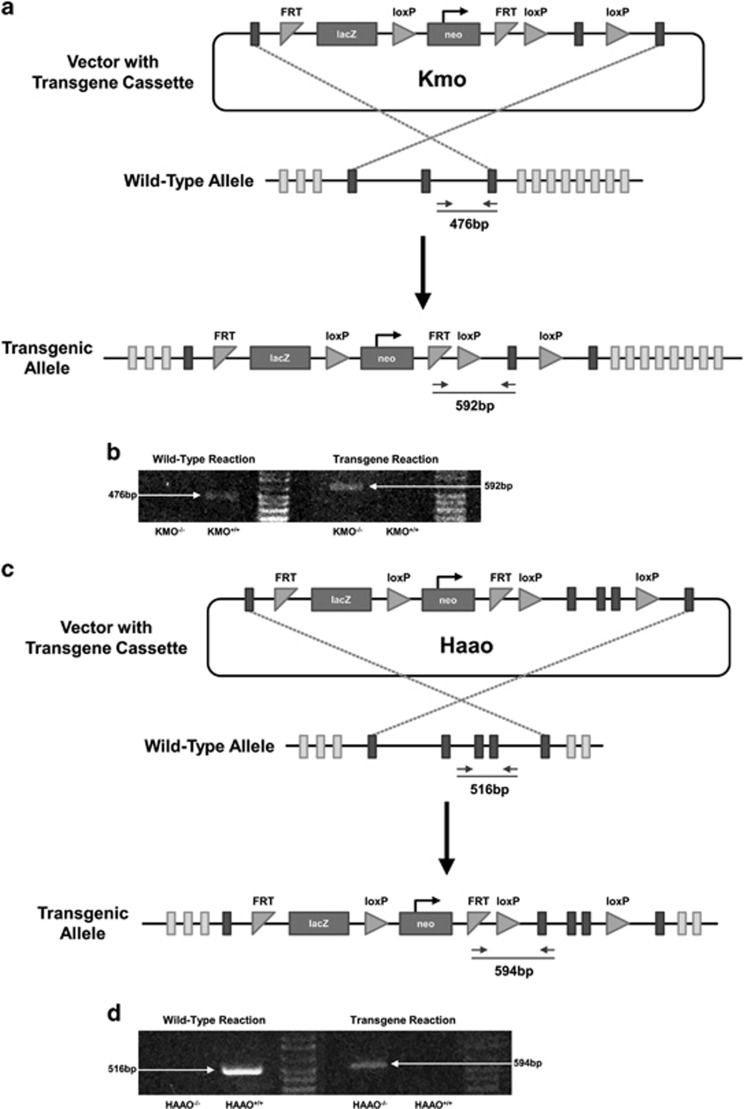
Kynurenine 3-monooxygenase (KMO) and 3-hydroxyanthranilic acid dioxygenase (HAAO) transgene construct. (**a**) The KMO transgene vector was designed with a neomycin (neo) selection cassette to test for site-specific integration of the transgene. Proper incorporation of the transgene results in gene-trapping between the reporter gene (lacZ) from the cassette and the Kmo gene during transcription. When these transcripts become spliced together, an insertion mutation is created resulting in a non-functional KMO protein and a KMO^−/−^ mouse. The vector also includes recombination sites (Flp recombination target (FRT), loxP) that can be used to create a conditional-ready mouse targeted for the gene of interest (that is, KMO-floxed). (**b**) The presence of the transgene in KMO transgenic mice were confirmed using RT-PCR for either the wild-type allele (476-bp band) or the transgenic allele (592-bp band), as indicated in **a**. (**c**) The HAAO transgene vector was designed identical to the KMO transgene (**a**), targeting the HAAO genetic sequence. (**d**) The presence of the HAAO transgene was confirmed in the same manner as the KMO transgene using a RT-PCR reaction for the wild-type allele (516 bp) and for the transgenic allele (594 bp).

Briefly, C57BL/6N mouse embryonic stem cells were injected with a vector containing a transgenic cassette directed for the target gene (Figures 2a and c). Stem cells were selected for site-specific integration of the transgene, microinjected into C57BL/6N mouse blastocysts and implanted into C57BL/6N female mice.^31^ During transcription, the proper incorporation of the transgene into the target gene results in gene-trapping between the reporter gene (lacZ) from the cassette and the endogenous gene.^32^ When these transcripts become spliced together, an insertion mutation is created resulting in a non-functional target protein. The presence of the Kmo transgene was confirmed by RT-PCR of genomic DNA using the following primers: 5′-ACCAGTCAGCAGGTCCTTGTTT-3′ (WT forward primer), 5′-CGCGTCGAGAAGTTCCTATTCC-3′ (Kmo transgene forward primer) and 5′-AACCCATGTTACCGTCACACAC-3′ (common reverse primer; Figure 2b). The Haao transgene was confirmed in the same manner using the following primers: 5′-GATAAGGGATTGGGGGTGTG-3′ (WT forward primer), 5′-GAAAGTATAGGAACTTCGTCGAGAT-3′ (Haao transgene forward primer) and 5′-GCCAAGGTCCTTACAGTGGA-3′ (common reverse primer; Figure 2d). Functional deletion of the target gene was confirmed using validated Taqman Gene Expression assays (Mm01321343_m1 (Kmo) and Mm00517945_m1 (Haao); Life Technologies, Grand Island, NY, USA) for real-time RT-PCR amplification of steady-state mRNA (data not shown). Upon confirmation of a knockout genotype, mice were back-crossed with C57BL/6J mice (Jackson Laboratory, Bar Harbor, ME, USA; stock# 000664) for five generations to maintain the in-house breeding colonies. C57BL/6J mice were used to supplement the WT littermate control group when needed, after confirming that there was not a significant difference in the phenotypic or metabolic responses to LPS (data not shown).

Three to five-month-old male mice were used for all experiments. *Ad libitum* food and water access was provided at all times. Two weeks before testing, mice were individually housed in a modified reverse light cycle (lights on 2300–1100 hours), gently handled and weighed each day.

### Treatments

LPS (*Escherichia coli*, serotype 0127:B8, Sigma-Aldrich, St Louis, MO, USA) was prepared in endotoxin-free saline on the morning of injections. LPS (0.5 mg kg^−1^) or saline was injected intraperitoneally (i.p.) 24 h before behavioral assessments and tissue collection.

3-HK (Sigma-Aldrich) was dissolved in 0.5 N HCl at a concentration of 4 ×, diluted to 2 × with 0.5 N NaOH and to 1 × with 0.2M (2 ×) phosphate-buffered saline. 3-HK was administered subcutaneously at 0.0, 3.33 or 10 mg kg^−1^ 30 min before behavioral testing or tissue collection, which was previously established as the time of peak brain concentrations following peripheral kynurenine administration.^33^

**Figure 3:**
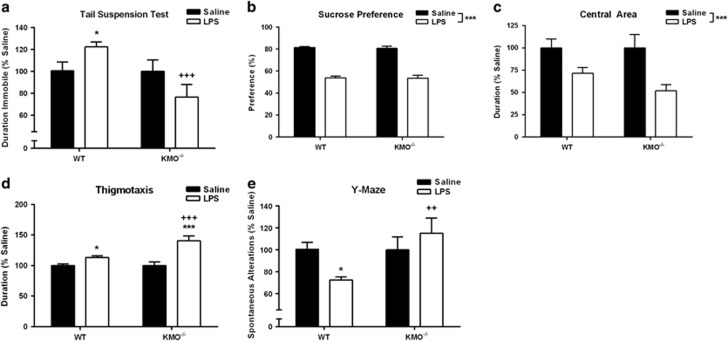
Kynurenine 3-monooxygenase (KMO) knockout mice are protected from distinct inflammation-induced depressive-like behaviors. (**a**) Twenty-four hours following injection with either lipopolysaccharide (LPS) or saline, wild type (WT) and KMO^−/−^ mice were tested in the tail suspension test (TST) to assess behavioral despair. LPS increases duration spent immobile (seconds, represented as % Saline) in WT mice but not in KMO^−/−^ mice. (**b**) Sucrose and water intake were recorded for the 24h following LPS or saline injections and were used to calculate sucrose preference (SP) to assess anhedonia-like behavior. LPS treatment resulted in a characteristic reduction in SP in both WT and KMO^−/−^ mice. (**c**) Similarly, at 24 h post treatment, duration (seconds, represented as % Saline) in the center of the open field (OF) was recorded as an index of anxiety-like behavior. (**d**) Duration spent near the walls of the OF or thigmotaxis behavior (seconds, represented as % Saline) was also recorded to asses anxiety-like behavior. In both OF assessments (**c**, **d**), LPS treatment resulted in similar elevations in anxiety-like behavior in WT and KMO^−/−^ mice. (**e**) Spontaneous alterations (represented as % Saline) between the three arms of a Y-maze were recorded as an index of working memory. LPS treatment resulted in a significant deficit in spontaneous alterations in WT mice but KMO^−/−^ mice were unaffected. Data represent sample means ± s.e.m. *n*=13–29 mice per group. *Main effect or *post hoc* comparison between saline and LPS within the same genotype. ^+^*Post hoc* comparison to WT with the same intraperitoneal (i.p.) treatment. ^*,+^*P*<0.05–0.01; ^**,++^*P*<0.01–0.001; ^***,+++^*P*<0.001.

**Figure 4:**
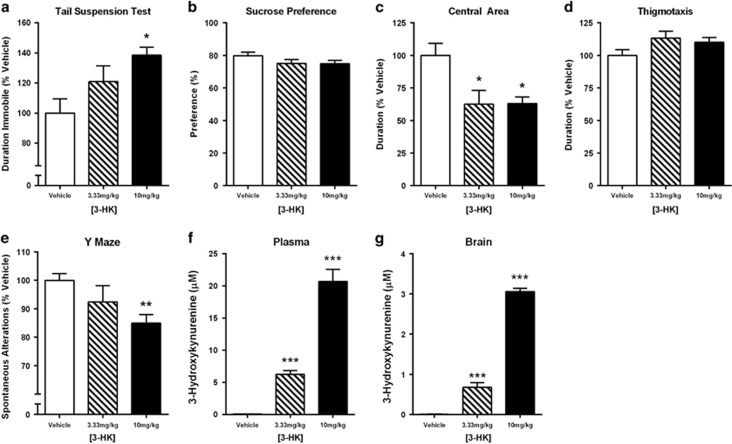
3-Hydroxykynurenine (3-HK) administration induces behavioral despair and working memory deficits. (**a**) The metabolite produced by kynurenine 3-monooxygenase (KMO), 3-HK, was injected subcutaneously 30 min before assessing behavioral despair in the tail suspension test (TST). 3-HK was administered at doses of 0.0 mg kg^−1^ (vehicle), 3.33 and 10 mg kg^−1^ and resulted in an increased immobile duration (seconds, represented as % Vehicle). (**b**) Sucrose preference (SP) was measured 2 h beginning 30 min post-subcutaneous 3-HK administration (0.0 mg kg^−1^ (vehicle), 3.33 and 10 mg kg^−1^), and anhedonia-like behavior was unaffected by peripheral 3-HK. Thirty minutes following 3-HK subcutaneous administration (0.0 mg kg^−1^ (vehicle), 3.33 and 10 mg kg^−1^), activity was recorded in the open field (OF) to determine anxiety-like behavior. 3-HK decreased the duration (seconds, represented as % Vehicle; (**c**) in the center of the OF while there was no impact of 3-HK on (**d**) thigmotoxis behavior. Working memory was also assessed in the Y-maze 30 min following subcutaneous administration (0.0 mg kg^−1^ (vehicle), 3.33 and 10 mg kg^−1^) of 3-HK. (**e**) Spontaneous alterations (% Vehicle) decreased following treatment with 3-HK. (**f**) Plasma 3-HK concentrations (μM) increased following administration of 3-HK. (**g**) Similarly, 3-HK (μM) assessed in brain tissue was elevated by peripheral 3-HK treatment. Data represent sample means ± s.e.m. *n*=5-16 mice per group. *Main effect or *post hoc* comparison to saline or vehicle. *P<0.05–0.01; **P<0.01–0.001; ***P<0.001.

**Figure 5:**
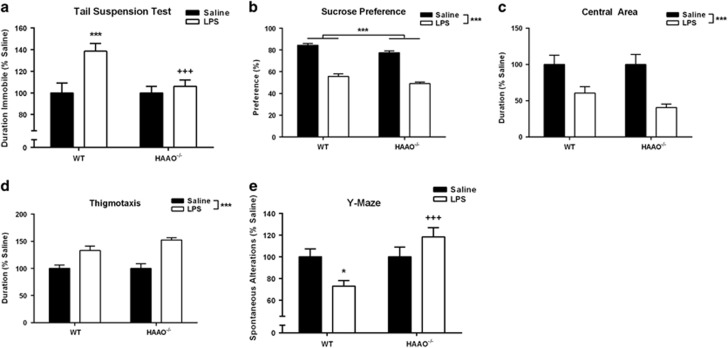
3-Hydroxyanthranilic acid dioxygenase (HAAO) transgenic mice are shielded from inflammation-induced behavioral despair. (**a**) HAAO^−/−^ or wild-type (WT) mice were assessed for duration spent immobile (s) in the TST 24 h following lipopolysaccharide (LPS) or saline injections. Whereas LPS treatment increased immobile duration (represented as % Saline) in WT mice, HAAO^−/−^ mice were protected from this LPS-induced change in behavior. (**b**) Following LPS treatment, both WT and HAAO^−/−^ mice had a reduction in sucrose preference (SP) representing anhedonia-like behavior. (**c**) In addition, behavior in the open field (OF) was analyzed for the effect of LPS treatment on duration spent in the center of the arena (represented as % Saline), an index of anxiety-like behavior. LPS reduced time spent in the central area similarly in both genotypes. (**d**) Further, thigmotaxis (duration) was extracted from the OF behavior (represented as % Saline), which was increased by LPS treatment both in WT and HAAO^−/−^ mice. (**e**) Spontaneous alternations (represented as % Saline) in a Y-maze, an assessment of working memory, were reduced by LPS treatment only in WT mice and not in HAAO^−/−^ mice. Data represent sample means ± s.e.m. and were analyzed using a two-analysis of variance (ANOVA), followed by the Holm–Sidak method for pairwise multiple comparisons. *n*=11–22 mice per group. *Main effect or *post hoc* comparison between saline and LPS within the same genotype. ^+^*Post hoc* comparison to WT with the same intraperitoneal (i.p.) treatment. ^*,+^*P*<0.05–0.01; ^**,++^*P*<0.01–0.001; ^***,+++^*P*<0.001.

*Treatment groups*: WT and KMO^−/−^ mice were treated (i.p.) with either saline (WT *n*=36; KMO^−/−^ *n*=24) or LPS (WT *n*=37; KMO^−/−^ *n*=24), followed by behavioral testing (Figure 3) and tissue collection (Table 1) at 24 h. WT mice were injected peripherally (subcutaneously) with 0.0 mg kg^−1^ (*n*=27), 3.33 mg kg^−1^ (*n*=17) or 10 mg kg^−1^ (*n*=20) 3-HK, followed by either behavior or tissue collection (Figure 4). WT and HAAO^−/−^ mice were also treated (i.p.) with either saline (WT *n*=26; HAAO^−/−^ *n*=29) or LPS (WT *n*=29; HAAO^−/−^ *n*=31), followed by behavioral assessment (Figure 5) and tissue collection (Table 1) at 24 h. Finally, WT mice were treated (i.p.) with either saline (*n*=12) or LPS (*n*=12) and brain regions were microdissected (Tables 2 and 3) at 24 h. Animals were randomly assigned to treatment groups within the appropriate genotype, and data were collected and analyzed by a treatment-blind observer. Previous studies conducted by our laboratory using similar treatments and behaviors were used to determine group sample size, which provide adequate effect size.^16, 33, 34^

### Behavioral testing

#### Locomotor activity and open field test

Exploratory locomotor activity and anxiety-like behavior were assessed 24 h post-LPS by placing mice in a dimly lit (~5 lux) 40 × 40 cm open field (OF) chamber for 5 min. Activity in the OF was video-recorded and assessed for total horizontal distance traveled and time spent in the central or outer areas using Ethovision XT 7.1 analysis software (Noldus, Leesburg, VA, USA) as previously described.^16^ The chamber was cleaned with 70% ethanol after each individual test.

#### Sucrose preference

Three days before LPS treatment, mice were trained using a two-bottle (water and 1% sucrose) testing paradigm. Following LPS injections, sucrose preference (SP) was assessed as an index of anhedonia-like behavior as previously described and calculated as (sucrose intake)/(water + sucrose intake) × 100.^16^ To control for potential place preference, bottles were placed on the outside edge of the wire food hopper and their relative location was alternated daily.

#### Tail suspension test

Immediately after the OF test, behavioral despair was measured in the tail suspension test (TST), as previously described.^14^ Immobility during the 6 min test was scored by a trained treatment-blinded observer. Any mice that climbed their tail, which occurred in fewer than 5% of experimental mice, were excluded from subsequent data analysis.

#### Y-Maze

Mice were placed in the maze and allowed to freely explore for 8 min. The movement and location of the mouse was recorded from an overhead camera, and the distance traveled and sequence of arm entries were scored from the video archive. An entry was scored only when the mouse's full body (minus tail) had entered 2-cm deep into the arm. A spontaneous alternation occurred when the mouse entered each of the three different arms sequentially before making a return entry to an already visited arm.

#### Behavioral timeline

Before treatment (saline or LPS i.p.), mice were trained on the two-bottle SP testing paradigm, and then SP data were collected during the 24 h following treatment and before behavioral testing. At 24 h following treatment, mice underwent testing in the OF and TST or the Y-maze immediately followed by tissue collection (as described below). For peripheral 3-HK experiments, mice were injected subcutaneously 30 min before either the TST, OF, Y-maze, a shortened 2 h SP test or tissue collection.

### Liquid chromatography/mass spectrometry

Either following behavior at ~25 h post-LPS treatment or 30 min following subcutaneous 3-HK administration, tissues were collected for analysis. Mice were killed by carbon dioxide asphyxiation and venous blood was collected into heparinized tubes for separation of plasma. Then, mice were immediately perfused with ice-cold heparinized sterile saline before collection of whole-brain or microdissection of dorsal hippocampus, ventral hippocampus, central amygdala and nucleus accumbens. Microdissections were conducted using a brain matrix (Stoelting, Wood Dale, IL, USA) and serial 1-mm coronal brain sections (based on stereological coordinates in a mouse brain atlas^35^). All tissue samples were stored at −80 °C until use. Samples were prepared for liquid chromatography/mass spectrometry (LC/MS) and analyzed for kynurenine metabolites as previously described.^20^ Briefly, thawed plasma samples were diluted with 5 × 0.2% acetic acid and 1 mM internal standards, transferred to Amicon Ultra filters (Millipore, Billerica, MA, USA) and centrifuged at 13 500 *g* for 1 hour at 4 °C. Frozen brain tissue was diluted with 3 × 0.2% acetic acid and 1 mM internal standards and then homogenized at 4 °C using an Omni International Bead Ruptor 24 Homogenizer (Kennesaw, GA, USA) with 1.4 mm zirconium ceramic oxide beads (Omni International) and settings of (pulse duration: 45 s, pulse number: 2, rest interval: 15 s). The brain homogenate was filtered as the plasma. LC/MS was performed on a Thermo Fisher Scientific Q Exactive mass spectrometer (Waltham, MA, USA) with online separation by a Thermo Fisher Scientific Dionex UltiMate 3000 HPLC and the resulting data were analyzed using Xcalibur 2.2 (Thermo Fisher Scientific) in the Mass Spectrometry Core Facility at the University of Texas Health Science Center at San Antonio. QA and 3-HAA were not reliably measured in microdissected brain region samples because of small sample size, nor was QA reliably detected in whole-brain samples.

### Statistical analysis

All statistical analyses were performed using SigmaPlot 12.0 (Systat Software, San Jose, CA, USA) and data represent group means ± s.e.m. Behavioral data are presented as %Saline (saline or LPS i.p.) or %Vehicle (3-HK subcutaneous) and were calculated based on saline within the individual genotype or on vehicle. Spurious data were identified as previously described^33^ and analyzed with either a one-tailed *t*-test, a one- or two-way analysis of variance. When a significant interaction was identified, *post hoc* analyses were completed using the Holm–Sidak method for pairwise multiple comparisons to identify significant differences between groups. Significant (*P*<0.05) within-group differences are denoted as such (*), whereas significant same treatment between-group differences are denoted as such (^+^).

## Results

### KMO mediates distinct inflammation-induced depressive-like behaviors

Mice were tested in the TST (Figure 3a) 24 h after i.p. LPS or saline, and a significant genotype × treatment interaction (*P*=0.009) was identified. WT mice exhibited a significant increase in duration spent immobile following LPS injections (*P*=0.029 versus saline), similar to previously observed LPS effects in the TST.^14^ However, LPS failed to cause an increase in the duration of immobility in KMO^−/−^ mice. LPS caused a significant reduction in SP (Figure 3b, *P*<0.001) that was not different between genotypes, and similarly the duration of time spent in the central area of an OF was significantly reduced by LPS-independent of genotype (Figure 2c, *P*<0.001). Thigmotaxis (Figure 3d), wall-hugging behavior, was increased after LPS in both WT (*P*=0.012) and KMO^−/−^ (*P*<0.001) mice, but to a greater extent in the KMO-deficient mice (*P*<0.001). Overall distance traveled in the OF was reduced modestly, but significantly, by LPS treatment (Supplementary Figure 1A, *P*<0.001) independent of genotype, as was LPS-induced reduction in body weight (Supplementary Figure 1B, *P*<0.001). A significant genotype × treatment interaction (F_1,23_=5.10, *P*=0.034) was apparent for spontaneous alternations during the Y-maze test (Figure 3e), where LPS reduced alternations in WT (*P*=0.046), but not in KMO^−/−^ mice.

### 3-HK dose-dependently induces a depressive-like behavioral phenotype

3-HK was administered subcutaneously 30 min before behavioral testing at the indicated doses. 3-HK induced a dose-dependent increase in the duration of immobility during the TST (Figure 4a, *P*=0.012). SP, measured during a 2-h probe beginning 30 min post injection, was unaffected by 3-HK administration (Figure 4b). The duration of time spent in the central area of an OF was significantly increased by 3-HK administration (Figure 4c, *P*=0.013); however, thigmotaxis behavior failed to reach significance (Figure 4c). Importantly, general locomotor activity in the OF, as evaluated by distance traveled, was not significantly affected by 3-HK administration (data not shown). Similar to TST immobility, exogenous 3-HK administration significantly reduced spontaneous alternations during the Y-maze test (Figure 4e, *P*=0.007) in a dose-dependent manner. Peripheral 3-HK administration dose-dependently increased 3-HK concentration in the plasma (Figure 4f, *P<*0.001) in line with behavioral changes observed. Similarly, central concentrations of 3-HK (Figure 4g) were dose-dependently elevated (*P<*0.001) in response to peripheral 3-HK injections.

### HAAO null mice are protected from peripheral LPS-induced behavioral despair

Twenty-four hours after LPS or saline treatment, depressive-like behaviors were assessed in HAAO^−/−^ or WT. LPS administration caused a 40% increase in the duration of immobility in WT mice in the TST; however, HAAO^−/−^ mice were entirely protected from this effect (Figure 5a, *P*=0.013). HAAO^−/−^ mice had similar LPS treatment responses as WT mice when assessed for anhedonia and anxiety-like behavior. Following LPS treatment, there was a significant decrease in SP caused by LPS (Figure 5b) in both HAAO^−/−^ and WT mice (*P*<0.001), and although no interaction was present, a modest but significant overall reduction in SP was apparent in HAAO^−/−^ mice (*P*<0.001). In the OF test, LPS caused a reduction in the duration of time spent in the central area (Figure 5c, *P*<0.001) and an increase in thigmotaxis behavior (Figure 5d, *P*<0.001); however, there was no significant effect of genotype. Similar to KMO^−/−^ mice, distance traveled during the OF (Supplementary Figure 1C) was reduced 24 h following LPS treatment independent of genotype (*P*<0.001), and LPS precipitated a reduction in body weight that was not different between genotypes (Supplementary Figure 1D, *P*<0.001). In addition, there was a significant genotype × treatment interaction in spontaneous alternations assessed in the Y-maze (Figure 5e, *P*=0.011), in which LPS treatment induced a reduction in WT mice (*P*=0.041) and not in HAAO^−/−^ mice.

### Characterization of brain and plasma kynurenine metabolism in KMO and HAAO knockout mice

Full statistical analysis is indicated in the table, and, for the sake of clarity, parsimonious reporting of significant effects are reported here. To determine the consequence of targeted deletions of *Kmo* and *Haao* genes on central kynurenine pathway metabolism (Figure 1), cerebral levels of tryptophan and kynurenine metabolites were measured in WT, KMO^−/−^ and HAAO^−/−^ mice 24 h after LPS or saline administration (Table 1). There was a significant main effect of both treatment and genotype on the concentration of central tryptophan (μM, Row 1). Consistent with previous findings,^20^ LPS treatment resulted in an elevation in central kynurenine levels (μM, Row 2) in WT, KMO^−/−^ and HAAO^−/−^ mice. However, there was a significant interaction between genotype and treatment as KMO^−/−^ mice had substantially higher brain kynurenine levels. 3-HK (μM, Row 3) was not detected in the brain of KMO^−/−^ mice, and was higher in HAAO^−/−^ mice relative to WT mice. However, there was a significant LPS-induced increase in 3-HK in WT mice, whereas there was no treatment effect in HAAO^−/−^ mice. Downstream of 3-HK, there was a significant interaction between genotype and treatment on the concentration of central 3-HAA (μM, Row 4). This effect is largely driven by the elevation of central 3-HAA in HAAO^−/−^ mice. QA was not reliably detected in any of the brain samples in our study. KA (μM, Row 6) was markedly elevated in KMO^−/−^ mice and decreased in HAAO^−/−^ mice, with no significant effect of LPS.

Plasma tryptophan levels (μM, Row 7) varied between genotype, but were significantly reduced by LPS in WT, KMO^−/−^ and HAAO^−/−^ mice. Kynurenine levels (μM, Row 8) were substantially higher in KMO^−/−^ mice overall. 3-HK (μM, Row 9) was detected in plasma of all mice, although near the lower limit of detection in KMO^−/−^ mice. LPS increased 3-HK levels in both WT and HAAO^−/−^ mice, but not in KMO^−/−^ mice. Plasma 3-HAA (μM, Row 10) was not detected in WT or KMO^−/−^ mice, and there was no LPS treatment effect in HAAO^−/−^ mice. QA levels (μM, Row 11) varied by genotype with no effect of treatment. Finally, there was a significant genotype × treatment interaction in the concentration of plasma KA (μM, Row 12). This effect was driven by a 25–30-fold increase in KA levels in the KMO^−/−^ mice. Overall, LPS reduced plasma KA levels.

### Kynurenine metabolism within discrete, behaviorally relevant, brain regions following peripheral immune challenge with LPS

Full statistical analysis is indicated in the table, and, for the sake of clarity, parsimonious reporting of significant effects are reported here. To determine whether region-specific differences in kynurenine metabolism underlie the distinct neurotoxic metabolism-dependent behavioral profile, brain region metabolite concentrations were determined 24 h post-saline or LPS treatment (Table 2). In the dorsal hippocampus, both tryptophan and kynurenine concentrations (μM) increased following LPS treatment. Neuroactive kynurenine metabolism also increased with elevations in 3-HK (μM), 3-HAA (μM) and XA (μM), whereas KA (μM) remained unchanged. XA has been reported to influence synaptic transmission as a metabotropic glutamate receptor group II ligand and a vesicular glutamate transporter inhibitor.^36, 37^ In the ventral hippocampus, LPS treatment also increased kynurenine and XA. However, no other metabolites were altered by LPS. In the central amygdala, tryptophan and kynurenine concentrations increased in response to LPS treatment. 3-HK, XA and KA were also significantly elevated following LPS treatment. In the nucleus accumbens, tryptophan and all kynurenine metabolites were increased following LPS treatment as in the central amygdala. 3-HAA was not detected in either the central amygdala or the nucleus accumbens.

To compare the relative LPS effect between brain regions, metabolite changes were normalized as % saline and analyzed (Table 3). There was a main effect of region on tryptophan concentration change following LPS treatment, whereby tryptophan was significantly higher in the central amygdala than the ventral or dorsal hippocampus. LPS-induced increases in kynurenine, 3-HK and XA were comparable across the different brain regions. Although 3-HAA was not detected in the central amygdala or nucleus accumbens, it was increased to a greater extent in dorsal compared with ventral hippocampus. There was also a significant effect of region on LPS-induced KA. This effect was driven by significantly greater LPS-induced increase in nucleus accumbens and central amygdala compared with hippocampal subregions.

## Discussion

The kynurenine pathway of tryptophan metabolism has been heavily implicated as a pathogenic factor in the development of depression, particularly within the context of inflammation-associated depression. We and others have demonstrated that IDO, the rate-limiting step of kynurenine metabolism (Figure 1), directly mediates the induction of depressive-like behaviors in response to peripheral immune challenge in preclinical models.^14, 16, 33^ However, the generation of neuroactive kynurenine metabolites occurs downstream of IDO.^20^ Whereas emergent clinical and preclinical data suggest that shifting the balance of downstream kynurenine metabolism to favor production of neurotoxic kynurenines drives depressive symptoms,^19, 20^ no mechanistic studies have been performed to directly test this hypothesis. Here, we utilized two novel transgenic mouse strains with targeted deletion of either KMO or HAAO (Figure 1) to determine whether neurotoxic kynurenine metabolism causes the development of depressive behaviors following peripheral immune activation with LPS (bacterial endotoxin). KMO^−/−^ mice do not generate detectible 3-HK in the brain before or after LPS challenge (Table 1). In contrast to inhibition of IDO, targeted deletion of KMO inhibited only a specific subset of LPS-induced depressive-like behaviors (Figure 3). Direct administration of 3-HK, the metabolic product of KMO, induced the development of depressive-like behaviors that were attenuated in the KMO^−/−^ mice (Figure 4). Although QA was not reliably detected in brain tissue (Table 1), a reduction in neurotoxic metabolism would presumably result in the generation of less QA. Therefore, LPS-induced depressive behavior was measured in HAAO^−/−^ mice, and these mice were also protected from the development of the same specific depressive-like behaviors (Figure 5). Together, these data confirm that KMO- and HAAO-dependent neurotoxic kynurenine metabolism directly mediates the development of inflammation-induced behavior despair and working memory deficits; behaviors regulated by hippocampal-dependent neurocircuitry.

Previous research in this area has suffered from an overall lack of functional precision, as studies that measured kynurenine metabolism downstream of IDO have evaluated only CSF in patients or whole-brain tissue in rodents.^19, 20^ For example, Raison *et al.*^19^ showed that the concentration of QA in the CSF was positively correlated with depression scores of controls and subjects receiving interferon immunotherapy.^19^ In addition, the first preclinical study to investigate the role of QA in driving depressive symptoms showed that LPS-induced depressive behaviors could be mitigated in inbred C57BL/6 mice by the NMDAR, ketamine, suggesting a pathogenic role for QA.^20^ Metabolic analysis in the study by Walker *et al.*^20^ indicated that LPS caused a shift toward neurotoxic metabolism in whole-brain tissue of a separate group of CD-1 outbred mice.^20^ Here, we report, for we believe the first time, direct evidence that directly implicates KMO-dependent kynurenine metabolism in LPS-induced depression. Further, as only specific behaviors were mitigated by genetic inhibition of neurotoxic kynurenine metabolism, the data suggested that kynurenine metabolism might differ between discrete brain regions. Indeed, Frenois *et al.*^38^ demonstrated that neuronal activation following peripheral LPS challenge of C57BL6/J mice (measured by FosB/Δ FosB immunohistochemistry) was not uniformly increased.^38^ Rather, discrete regions with known relevance to depressive behavioral domains were more strongly increased, including hippocampus, nucleus accumbens and central amygdala.^38^

Whereas a previous report by Giorgini *et al.*^39^ characterized the metabolic changes that occur in response to a KMO deficiency in a separate strain of mice,^39^ the data in Figure 3 are the first to demonstrate the significance of a KMO genetic deletion on the behavioral consequences of peripheral LPS administration. KMO^−/−^ mice have disrupted central and peripheral kynurenine metabolism (Table 1) in a similar pattern to this previous study.^39^ Interestingly, baseline kynurenine levels are significantly elevated in KMO^−/−^ mice without any apparent changes to baseline depressive-like phenotype. This is seemingly in contrast to previous data from our laboratory, and others demonstrating that direct administration of kynurenine can induce deficits in novel object recognition and spatial working memory alter fear conditioning and induce anhedonia.^16, 33, 40, 41, 42^ It is possible that the accumulation of kynurenine over time through development allows the system to adjust preventing any potential behavioral changes. Peripheral LPS treatment in KMO^−/−^ mice precipitates an increase in central kynurenine without any changes in central 3-HK. The metabolite analyses from HAAO^−/−^ mice (Table 1) are the first from a genetic mutant model targeted at this enzyme that metabolizes 3-HAA to QA. Although QA was not reliably detected in any brain tissue sample, both central 3-HK and 3-HAA in HAAO^−/−^ mice were increased over WT levels, as would be predicted in mice lacking the enzyme to metabolize 3-HAA to QA. In the plasma, 3-HK and 3-HAA were similarly elevated in HAAO^−/−^ mice; however, QA was still detectable, indicating the potential for an alternate metabolic source of QA. Although surprising, this peripheral QA is unable to cross the blood–brain barrier and therefore has no direct impact on central QA concentrations and does not contribute to the behavioral phenotype of HAAO^−/−^ mice.

It is well accepted that depressive symptoms occur across multiple behavioral domains, and that distinct regions of the brain are critical to each of the various domains of depressive-like behavior. In fact, this is the basic tenet of the National Institute of Mental Health's (NIMH) Research Domain Criteria (RDoC, http://www.nimh.nih.gov/research-priorities/rdoc/index.shtml) framework to guide mental health research.^43, 44^ Preclinical neuropsychiatric research necessarily employs an RDoC framework that deconstructs a behavioral phenotype (neuropsychiatric disorders being modeled) into core behaviors (analogous to symptoms) that are functionally controlled by primary neurocircuits in discrete brain regions. To determine whether increased neurotoxic kynurenine metabolism generates the metabolic substrates to cause the distinct depressive-like behaviors observed in Figures 3 and 5, kynurenine metabolites were measured in the dorsal hippocampus, ventral hippocampus, central amygdala and nucleus accumbens of C57BL6/J mice (Table 2), the same background strain of the KMO^−/−^ and HAAO^−/−^ mice, at 24 h post treatment. These regions were chosen for their relevance to depression in humans as well as the individual behaviors analyzed in this study following peripheral inflammation.^45, 46, 47, 48^ Immobility during the TST and spontaneous alternations in Y-maze are behaviors known to require hippocampal function, and their analogous depressive symptoms are behavioral despair and working memory.^49, 50, 51^ However, SP requires reward circuitry of the nucleus accumbens, and reduced preference reflects an anhedonia-like phenotype.^46^ Behavior in the OF reflects anxiety-like states that involve amygdala activity.^52, 53^

Consistent with previous studies, LPS caused a reduction in circulating tryptophan levels and a robust increase in the concentration of kynurenine in both the periphery and in each brain region analyzed (Tables 1 and 2). However, an interesting pattern was noted in the metabolic profile downstream of kynurenine. Neurotoxic metabolites were generally increased across each brain region, but KA was also significantly increased in central amygdala and nucleus accumbens. Only the dorsal hippocampus reflected a clear shift toward neurotoxic and neuromodulatory (KMO-dependent) metabolism without a parallel increase in the opposing KAT-dependent metabolic branch. The specific behaviors known to involve hippocampal activity were mitigated by genetic inhibition of neurotoxic kynurenine metabolism (in KMO^−/−^ and HAAO^−/−^ mice) and precipitated by direct administration of 3-HK. The hippocampus was the only discrete brain region where LPS treatment resulted in a clear and unbalanced shift toward the generation of KMO-dependent metabolites. These findings are consistent with the contemporary hypothesis that disrupting the metabolic balance of the kynurenine pathway, rather than simply increasing overall kynurenine metabolism, defines the functional pathogenic context by which kynurenine metabolism mediates the behavioral effects of inflammation.^27, 28^ Further, these data support recent findings from our laboratory, demonstrating that peripheral LPS induces brain region-dependent changes in the balance of kynurenine metabolites, specifically favoring neurotoxic metabolite production in the hippocampus.^34^ Recent data characterizing the regional characteristics of microglia throughout the brain demonstrated that not all microglia express the same functional expression markers.^54^ Specifically, it was noted that microglia in the hippocampus were more responsive to pro-inflammatory stimuli, earning them the label ‘immunovigilant'.^54^ This hyper-responsive state of hippocampal microglia could underlie the region-specific elevation in neurotoxic kynurenine metabolism described in Table 2. Recent clinical data demonstrated that, following a suicide attempt in depressed patients, CSF QA remains elevated while CSF KA stayed decreased.^55^ In patients with mastocytosis, associated with comorbid depression and inflammation, plasma QA and plasma KA were found to be elevated.^56^ More specifically, in depressed patients, the serum KA/QA ratio (neuroprotective ratio) was positively correlated with increasing volumes of the hippocampus and amygdala.^57^

Previous data in mice with either genetic or systemic inhibition of IDO clearly indicate a pivotal role for kynurenine metabolism in mediating depressive-like behaviors in response to peripheral immune challenge. The general increase in kynurenine metabolism across each brain region (Table 2) that was identified in this study supports the conclusions of a broad importance of IDO in mediating the effects of LPS. However, targeting IDO does not provide any indication of which neuroactive downstream metabolites are responsible for driving inflammation-induced behavioral phenotypes. Whereas the previous study by Walker *et al.*^20^ found that both LPS-induced increases in immobility and reduction in SP could be mitigated by the NMDAR antagonist, ketamine, a direct pathogenic role for QA action on NMDARs can only be speculated.^20^ Here, we provide the first direct evidence that a shift toward neurotoxic metabolism in the hippocampus mediates the behavioral deficits following peripheral LPS. In contrast to the data from Walker *et al.,*^20^ we found that LPS-induced reductions in SP were not KMO- or HAAO-dependent, as their data suggested. This discrepancy could be the result of strain-related differences in LPS-induced metabolism of CD-1 versus C57BL6/J mice, or more likely other neuroactive kynurenines are responsible for driving the LPS-induced anhedonia phenotype, such as XA or KA. It is also possible that the chronic alterations in kynurenine metabolites that exist in the conventional KMO and HAAO null mice results in a compensatory recalibration of the reward system, as the effects of acute and subchronic elevations in KA on the dopaminergic system are well established.^58, 59^ Although no baseline differences in SP were observed, it is of interest to note that the LPS-stimulated increase in nucleus accumbens KA could locally decrease extracellular dopamine, similar to a previously demonstrated effect of exogenous KA application to the striatum.^60^ This local reduction in extracellular DA could contribute to the development of anhedonia behavior following LPS treatment.

Direct administration of exogenous kynurenine has been utilized in a number of contexts that result in behavioral changes.^40, 41, 59^ Our previous work demonstrated that exogenous kynurenine acutely precipitates a depressive-like phenotype similar to LPS.^16^ Chronic administration of kynurenine during early development or to adult mice has also been used to elevate KA levels in the brain, resulting in deficits in sensorimotor gating, attentional processing of environmental stimuli, spatial working memory and contextual learning memory.^40, 41, 61^ The data here are the first to demonstrate that exogenously administered peripheral 3-HK is able to precipitate the development of behavioral despair in the TST, impair working memory in the Y-maze and induce anxiety-like behavior assessed in the OF. Of note, general locomotor activity in the OF (Supplementary Figure 1A, 1C), although confirmatory of a positive effect of LPS, was not correlated to depressive-like behaviors in any of the testing paradigms. This effect of LPS in the OF is similar to previously published data from our laboratory, demonstrating that duration in the central area (anxiety-like behavior) does not depend on locomotor activity.^16^ Although the open was conducted under low-light conditions and more light might provide a greater ethological conflict for assessing anxiety-like behavior, the primary purpose of this assessment was to determine the impact of treatment on locomotor activity.

Together, these data significantly enhance our understanding of the metabolic substrates that mediate inflammation-induced depressive-like behavior. Whereas a majority of brain kynurenine is supplied by the circulation,^62^ these data indicate that downstream metabolism is a regionally regulated process with implications for behavioral pathology. Our data indicate that hippocampal-dependent behaviors may be particularly vulnerable to neurotoxic dysregulation of kynurenine metabolism, which could also have larger implications in the context of neurodegenerative disease and chronic inflammatory conditions. Therapeutic manipulation of the kynurenine metabolic pathway remains a potentially valuable strategy for alleviating depressive symptoms, particularly in the context of inflammation and comorbidity.

## Figures and Tables

**Table 1:** KMO and HAAO knock-out mice brain and plasma kynurenine metabolite analysis following peripheral inflammation

	*Genotype*						*Main effects*		*Interaction*
	*WT*		*KMO*^*−/−*^		*HAAO*^*−/−*^		*Treatment* P*-value*	*Genotype* P*-value*	*Treatment × genotype*
	*Saline*	*LPS*	*Saline*	*LPS*	*Saline*	*LPS*			
*Brain metabolites (μM)*									
Tryptophan	31.20 (2.1)	34.44 (2.9)	37.54 (8.0)	45.25 (8.0)	55.49 (3.3)	67.96 (0.9)	*P*<0.05	*P*<0.001	n.s.
Kynurenine	0.17 (0.01)	0.59 (0.06)**	2.95 (0.2)^+++^	3.82 (0.3)^***,+++^	0.32 (0.05)	2.02 (0.1)^***,+++^	*P*<0.001	*P*<0.001	*P*<0.001
3-HK	0.044 (0.007)	0.17 (0.03)***	n.d.	n.d.	0.54 (0.2)	0.51 (0.08)	n/a	n/a	n/a
3-HAA	0.29 (0.06)	0.25 (0.04)	0.26 (0.03)	0.12 (0.04)	0.99 (0.07)^+++^	0.44 (0.05)^***,++^	*P*<0.001	*P*<0.001	*P*<0.001
QA	n.d.	n.d.	n.d.	n.d.	n.d.	n.d.	n/a	n/a	n/a
KA	0.12 (0.04)	0.10 (0.02)	0.37 (0.08)	0.31 (0.05)	0.021 (0.002)	0.038 (0.001)	n.s.	*P*<0.001	n.s.

*Plasma metabolites (μM)*									
Tryptophan	96.00 (4.7)	78.36 (7.4)	65.72 (3.9)	49.39 (2.6)	120.62 (9.5)	98.23 (3.0)	*P*<0.05	*P*<0.001	n.s.
Kynurenine	0.80 (0.06)	2.41 (0.1)	11.13 (2.0)	9.86 (0.3)	0.88 (0.08)	3.19 (0.4)	n.s.	*P*<0.001	n.s.
3-HK	0.083 (0.01)	0.45 (0.03)***	0.0098 (0.002)	0.017 (0.006)^+++^	0.13 (0.02)	0.87 (0.08)^***,+++^	*P*<0.001	*P*<0.001	*P*<0.001
3-HAA	n.d.	n.d.	n.d.	n.d.	2.17 (0.27)	3.16 (1.0)	n/a	n/a	n/a
QA	0.39 (0.03)	0.48 (0.08)	0.24 (0.008)	0.30 (0.004)	0.64 (0.04)	0.58 (0.02)	n.s.	*P*<0.001	n.s.
KA	0.94 (0.1)	0.59 (0.07)	31.45 (5.3)^+++^	15.78 (0.8)^***,+++^	0.24 (0.04)	0.21 (0.02)	*P*<0.001	*P*<0.001	*P*<0.001

Abbreviations: HAAO, 3-hydroxyanthranilic acid dioxygenase; i.p., intraperitoneal; KA, kynurenic acid; KMO, kynurenine 3-monooxygenase; LC/MS, liquid chromatography/mass spectrometry; LPS, lipopolysaccharide; n/a, statistical analysis not applicable; n.d., metabolite concentrations not reported because of high number of samples that were not detected with the LC/MS method utilized to analyze the samples; n.s., no significant difference; QA, quinolinic acid; WT, wild type; 3-HAA, 3-hydroxyanthranilic acid; 3-HK, 3-hydroxykynurenine.

Kynurenine metabolites (tryptophan, kynurenine, KA, 3-HK, 3-HAA and QA) were quantified (μM) by LC/MS in the plasma and whole-brain samples from WT, KMO^−/−^ and HAAO^−/−^ mice following i.p. treatment with saline or LPS (0.5 mg kg^−1^). Concentration values represent sample mean (s.e.m.). *n*=4–15 samples per group.

*Main effect or *post hoc* comparison between saline and LPS within the same genotype.

^+^*Post hoc* comparison with WT with the same i.p. treatment

.

^*,+^*P*<0.05–0.01; ^**,++^*P*<0.01–0.001; ^***,+++^*P*<0.001.

**Table 2:** Brain region kynurenine metabolite analysis following peripheral lipopolysaccharide

*Brain region*	*Metabolite (μM)*	*Treatment (i.p.)*		t-*Test*,P-*value*
		*Saline*	*LPS*	
Dorsal hippocampus	Tryptophan	102.93 (5.1)	129.83 (4.1)***	*P*<0.001
	Kynurenine	0.39 (0.03)	1.02 (0.1)***	*P*<0.001
	3-HK	0.13 (0.01)	0.19 (0.02)*	*P*<0.05
	3-HAA	1.35 (0.06)	1.86 (0.1)**	*P*<0.01
	KA	0.095 (0.02)	0.13 (0.02)	n.s.
	XA	0.0085 (0.001)	0.023 (0.003)**	*P*<0.01
				
Ventral hippocampus	Tryptophan	95.38 (4.5)	122.83 (9.2)	n.s.
	Kynurenine	0.33 (0.04)	0.85 (0.04)***	*P*<0.001
	3-HK	0.12 (0.02)	0.15 (0.02)	n.s.
	3-HAA	1.62 (0.08)	1.60 (0.2)	n.s.
	KA	0.12 (0.01)	0.14 (0.03)	n.s.
	XA	0.012 (0.001)	0.024 (0.003)*	*P*<0.05
				
Central amygdala	Tryptophan	119.34 (5.7)	218.78 (17.0)***	*P*<0.001
	Kynurenine	0.46 (0.04)	1.60 (0.1)***	*P*<0.001
	3-HK	0.11 (0.01)	0.19 (0.03)*	*P*<0.05
	3-HAA	n.d.	n.d.	n/a
	KA	0.12 (0.02)	0.40 (0.1)*	*P*<0.05
	XA	0.0076 (0.002)	0.015 (0.002)**	*P*<0.01
				
Nucleus accumbens	Tryptophan	112.82 (9.8)	167.35 (19.3)*	*P*<0.05
	Kynurenine	0.45 (0.07)	1.59 (0.3)**	*P*<0.01
	3-HK	0.14 (0.02)	0.24 (0.02)**	*P*<0.01
	3-HAA	n.d.	n.d.	n/a
	KA	0.20 (0.04)	0.78 (0.2)**	*P*<0.01
	XA	0.0073 (0.002)	0.016 (0.002)*	*P*<0.05

Abbreviations: i.p., intraperitoneal; KA, kynurenic acid; LC/MS, liquid chromatography/mass spectrometry; LPS, lipopolysaccharide; n/a, statistical analysis not applicable; n.d., metabolite concentrations not reported because of high number of samples that were not detected with the LC/MS method utilized to analyze the samples; n.s., no significant difference; XA, xanthurenic acid; 3-HAA; 3-hydroxyanthranilic acid; 3-HK, 3-hydroxykynurenine.

Kynurenine metabolites (tryptophan, kynurenine, KA, 3-HK and 3-HAA) were quantified (μM) by LC/MS in relevant brain regions (dorsal hippocampus, ventral hippocampus, central amygdala and nucleus accumbens) following i.p. treatment with saline or LPS (0.5 mg kg^−1^). Concentration values represent sample mean (s.e.m.) and were analyzed using a *t*-test or the Mann–Whitney rank sum test, if the data failed testing for equal variance or normality. *n*=6–12 samples per treatment

.

Significant differences are reported as: *P<0.05–0.01; **P<0.01–0.001; ***P<0.001.

**Table 3:** Brain region metabolite analysis following peripheral inflammation (% saline)

*Metabolite (% saline)*	*Brain region*				*ANOVA*,P-*value*
	*Dorsal hippocampus*	*Ventral hippocampus*	*Central amygdala*	*Nucleus accumbens*	
Tryptophan	126.14 (4.2)	128.78 (10.1)	183.33 (15.1)	148.33 (18.0)	*P*<0.01
Kynurenine	259.03 (19.8)	254.37 (14.6)	344.91 (26.7)	356.87 (70.5)	n.s.
3-HK	146.92 (16.5)	127.98 (14.7)	170.49 (25.2)	169.22 (15.0)	n.s.
3-HAA	137.72 (11.1)	99.30 (12.6)	n.d.	n.d.	*P*<0.05
KA	133.54 (20.1)	109.07 (25.9)	323.49 (110.3)	391.40 (93.6)	*P*<0.01
XA	268.78 (37.2)	201.84 (28.4)	200.66 (29.0)	224.32 (38.5)	n.s.

Abbreviations: ANOVA, analysis of variance; i.p., intraperitoneal; KA, kynurenic acid; LC/MS, liquid chromatography/mass spectrometry; LPS, lipopolysaccharide; n.d., metabolite concentrations not reported because of high number of samples that were not detected with the LC/MS method utilized to analyze the samples; n.s., no significant difference; XA, xanthurenic acid; 3-HAA; 3-hydroxyanthranilic acid; 3-HK, 3-hydroxykynurenine.

Kynurenine metabolites (tryptophan, kynurenine, KA, 3-HK and 3-HAA) were quantified (μM) by LC/MS in relevant brain regions (dorsal hippocampus, ventral hippocampus, central amygdala and nucleus accumbens) following i.p. treatment with saline or LPS (0.5 mg kg^−1^). Concentration values were then used to calculate % saline values, which are presented in the table as mean (s.e.m.). *n*=6–12 samples per treatment

.

Significant differences are reported as: *P<0.05–0.01; **P<0.01–0.001; ***P<0.001.
