# Supplementary material for: Neurotoxic kynurenine metabolism is increased in the dorsal hippocampus and drives distinct depressive behaviors during inflammation
Source: Transl Psychiatry. 2016 Oct 18;6(10):e918–. doi: 10.1038/tp.2016.200 (PMC5315555; doi:10.1038/tp.2016.200)
Supplement: Supplementary Figure Legend [file tp2016200x1.docx]

**Supplemental Figure Legend**

Supplemental Figure 1. Open field locomotor activity and change in body weight. (A, C) Locomotor activity was recorded as distance traveled in the OF (cm, represented as % Saline) 24h following LPS treatment. (B, D) Body weight was recorded prior to and following treatment then used to calculate change in grams (Δg) over the 24h treatment period. (A) LPS treatment caused a similar reduction in locomotor activity in WT and KMO^-/-^ mice. (B) WT and KMO^-/-^ mice experienced a decrease in body weight in the 24h following treatment with LPS. (C) WT and HAAO^-/-^ mice had a similar reduction in locomotor activity following LPS treatment. (D) LPS treatment resulted in a similar decrease in body weight in WT and HAAO^-/-^ mice. Data represent sample means ± SEM. n = 5-29 mice/group.

* = main effect or post-hoc comparison between saline and LPS within the same genotype

+ = post-hoc comparison to WT with the same i.p. treatment

*,^+^ p<0.05-0.01 **,^++^ p<0.01-0.001 ***,^+++^ p<0.001
