# Supplementary figures and images for: Neurotoxic kynurenine metabolism is increased in the dorsal hippocampus and drives distinct depressive behaviors during inflammation
Source: Transl Psychiatry. 2016 Oct 18;6(10):e918–. doi: 10.1038/tp.2016.200 (PMC5315555; doi:10.1038/tp.2016.200)

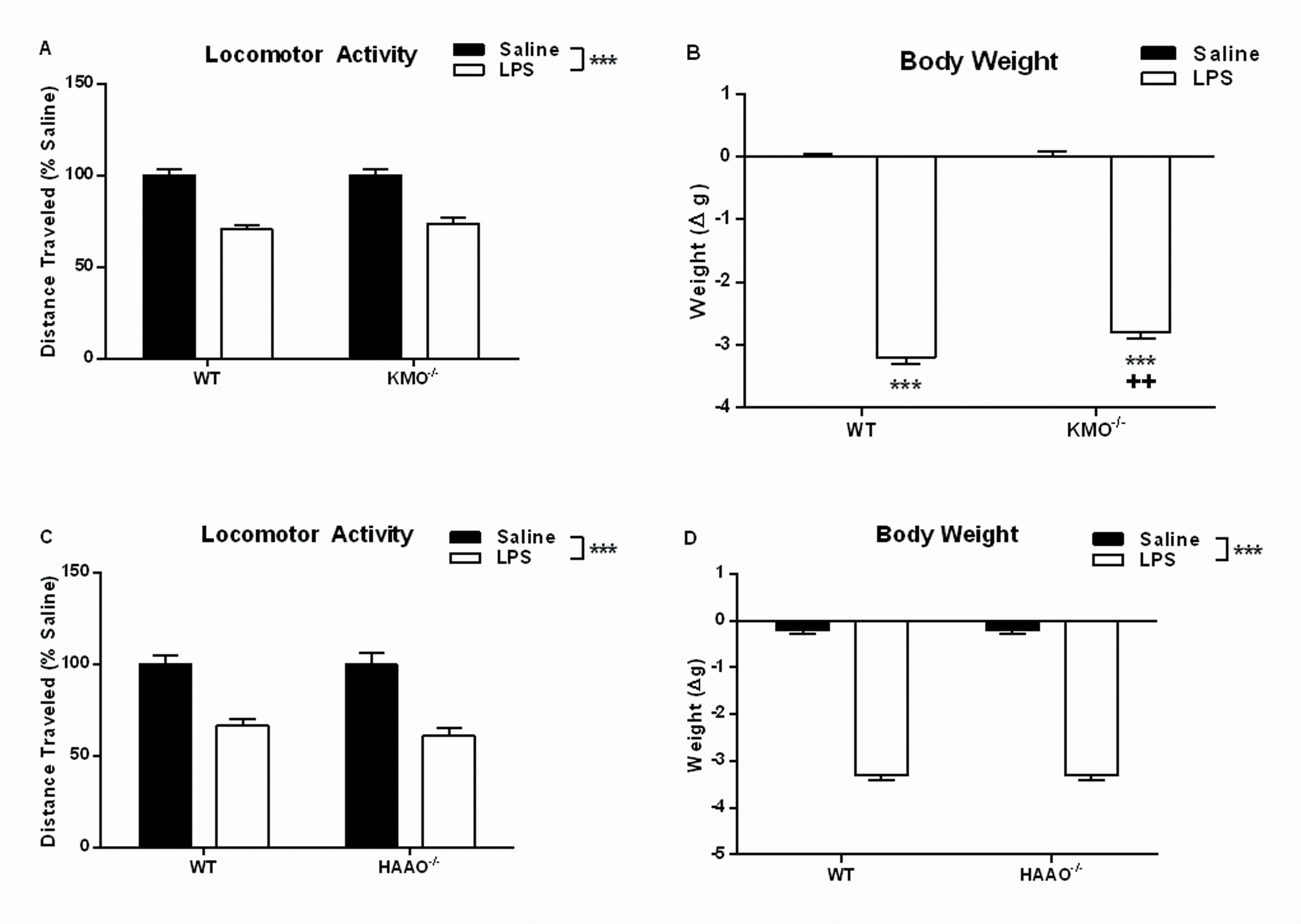

Supplement: Supplementary Figure 1 [file tp2016200x2.tif]
